# Factors influencing sleep disorders in perimenopausal women: a systematic review and meta-analysis

**DOI:** 10.3389/fneur.2025.1460613

**Published:** 2025-02-07

**Authors:** Weisi Zeng, Jialan Xu, Ying Yang, Meiling Lv, Xin Chu

**Affiliations:** School of Nursing, Chengdu University of Traditional Chinese Medicine, Chengdu, Sichuan, China

**Keywords:** perimenopause, menopause, climacteric, sleep disturbance, insomnia, sleep disorder

## Abstract

**Background:**

To determine the influencing factors of sleep disorders in perimenopausal women by Meta-analysis.

**Methods:**

A comprehensive literature search was conducted by PubMed, Embase, CINAHL, and Web of Science(from inception to December 1,2023). Two researchers independently performed literature screening, quality evaluation and data extraction, and Stata16.0 software were used for Meta-analysis.

**Results:**

A total of 12 studies involving 11,928 perimenopausal women with sleep disorders were included. The results of Meta-analysis showed that depression(OR = 2.73, 95%CI 1.65 ~ 4.52), hot flashes (OR = 2.70, 95%CI 1.81 ~ 4.02), chronic disease (OR = 1.39, 95%CI 1.24 ~ 1.56) and psychotropic drug use(OR = 3.19, 95%CI 1.31 ~ 7.77) were risk factors for sleep disorders in perimenopausal women (*p* < 0.05).

**Conclusion:**

Sleep disorder is one of the most common symptoms in perimenopausal women, and its influencing factors should be paid attention to. Healthcare managers can further improve and standardize the prevention and management of sleep disorders in perimenopausal women according to the influencing factors, accurately identify high-risk groups, implement intervention measures, and reduce the severity and incidence of sleep disorders in perimenopausal women.

## 1 Introduction

Perimenopause refers to the last years of a woman’s reproductive period, from the onset of menstrual irregularity until the end of amenorrhoea 1 year later (1), perimenopause is a physiological transition period for women. Due to the gradual decline of ovarian function and the decline of estrogen levels, women will have a variety of clinical symptoms such as insomnia, hot flashes, palpitations, menstrual disorders, irritability, osteoporosis, etc.

Sleep disorders have been cited as one of the top health concerns of menopausal women (2), and one of the most common symptoms (3, 4). Sleep disturbances are broadly defined as changes in the quality, quantity and timing of sleep that affect quality of life and daily functioning, with an estimated prevalence of 50–55%. Patients were diagnosed with insomnia if they complained of sleep problems (insomnia at onset or maintenance) and other daytime symptoms (such as mood disorders, lethargy, or decreased attention) at least 3 nights a week for more than 3 months. The International Classification of Sleep Disorders-Third Edition (ICSD-3) combines all insomnia diagnoses (primary and comorbid)into a single insomnia disorder (5). Although there are several subtypes of insomnia (idiopathic, psychophysiological, and paradoxical), the diagnosis and treatment are similar (6).

Studies have shown that the incidence of sleep disorders in perimenopausal women are 1.3–1.6 times that of premenopausal women (7), which are significantly higher than the incidence of insomnia in other age groups (8–10). As well as affecting mental health, sleep disorders contribute to morbidity and mortality from neurocognitive and cardiometabolic diseases and increase healthcare costs (11–13). In addition, perimenopausal sleep disturbances are often associated with other disorders such as weight loss/gain, depression, hot flushes, fatigue, reduced/decreased appetite and nocturia (14, 15), amplifying the harm of insomnia. Prolonged poor sleep can have a serious impact on women’s health and well-being (16, 17), placing them at risk for chronic diseases including diabetes, heart disease, obesity, and depression (18, 19). To our knowledge, no study has thoroughly and comprehensively analyzed the different factors that affect sleep quality in perimenopausal women. Therefore, this study aimed to provide updated evidence on factors contributing to sleep disturbances in menopausal women using a meta-analysis of cross-sectional, cohort, and case–control studies in perimenopausal women, contributing to the further data to develop an effective sleep disorder prevention programs for menopausal women with sleep disorders.

## 2 Methods

This meta-analysis was completed in accordance with the Preferred Reporting Items for Systematic Reviews and Meta-Analyses statement (20) and was registered with PROSPERO (Registration NO: CRD42023479749).

### 2.1 Search strategy

Studies delineating the influencing factors or risk factors for sleep disorders in perimenopausal women were searched using medical specialty databases of PubMed, Web of Science, Embase, CINAHL. The retrieval time was from the establishment of the database to December 2023. In addition, the references of the included literature were traced back to supplement the acquisition of relevant literature. Retrieve take subject and words combination of freedom. Search terms include: perimenopause, menopause, climacteric, insomnia, sleep disorder, Influence factor, etc. PubMed, for example, the specific retrieval strategies are shown in Table 1.

**Table 1:** Search strategy used in PubMed database.

Number	Search terms
#1	“perimenopause”[MeSH]OR“perimenopause”[Title/Abstract] OR “perimenopause syndrom”[Title/Abstract]OR “perimenopause symptoms”
#2	“menopause” [MeSH] OR “menopause” [Title/Abstract] OR “menopause syndrome” [Title/Abstract] OR “menopause symptoms”[Title/Abstract]
#3	“climacteric”[MeSH]OR “climacteric”[Title/Abstract]OR “climacteric syndrome”[Title/Abstract]OR “climacteric symptoms”[Title/Abstract]
#4	“postmenopause”[MeSH]OR“postmenopause”[Title/Abstract]
#5	#1 OR #2 OR #3 OR #4
#6	“insomnia”[MeSH]OR “insomnia”[Title/Abstract]
#7	“sleep disorder”[MeSH]OR “sleep disorder”[Title/Abstract]
#8	#6 OR #7
#9	“influencing factor”[Title/Abstract] OR “influence factor”[Title/Abstract]OR “risk factor”[Title/Abstract]OR“relevant factor”[Title/Abstract]
#10	#5 AND #8 AND #9

### 2.2 Inclusion and exclusion criteria

#### 2.2.1 Inclusion criteria

(a) Literature type: cross-sectional study, case–control study or cohort study; (b) Subjects: perimenopausal and women with sleep disorders, excluding primary sleep disorders. The duration of disease, age, and race of the subjects were not limited. The diagnostic criteria of perimenopause were based on the diagnostic criteria of the International Menopause Society in 2011 and the North American Menopause Society in 2001. Sleep disorders were diagnosed according to the International Classification and Diagnostic Criteria of Sleep Disorders developed by the American Academy of Sleep Medicine; (c) Outcome indicators: influencing factors or risk factors of sleep disorders in perimenopausal women, with clear evaluation methods and measurement standards. The original study has odds ratio (OR) and 95% confidence interval (CI) or documents that can convert a logistical regression assessment into a standard error of the logistic regression coefficient.

#### 2.2.2 Exclusion criteria

(a) Biological research or animal experiments; (b) Review, systematic review, meta-analysis, conference, case reports, dissertation of degree; (c) The disease under study was not perimenopausal; (d) Subjects underwent non-natural means of menopause such as hysterectomy or oophorectomy, or have serious health problems known to compromise ovarian function; (e) Study where relevant data cannot be extracted or data were incomplete; (f) No comparability of the groups; or unclear definition of influencing factors; (g) Full text study cannot be obtained; (h) Repeated publication of literature; (i) The language is not English.

### 2.3 Data extraction

**Figure 1:**
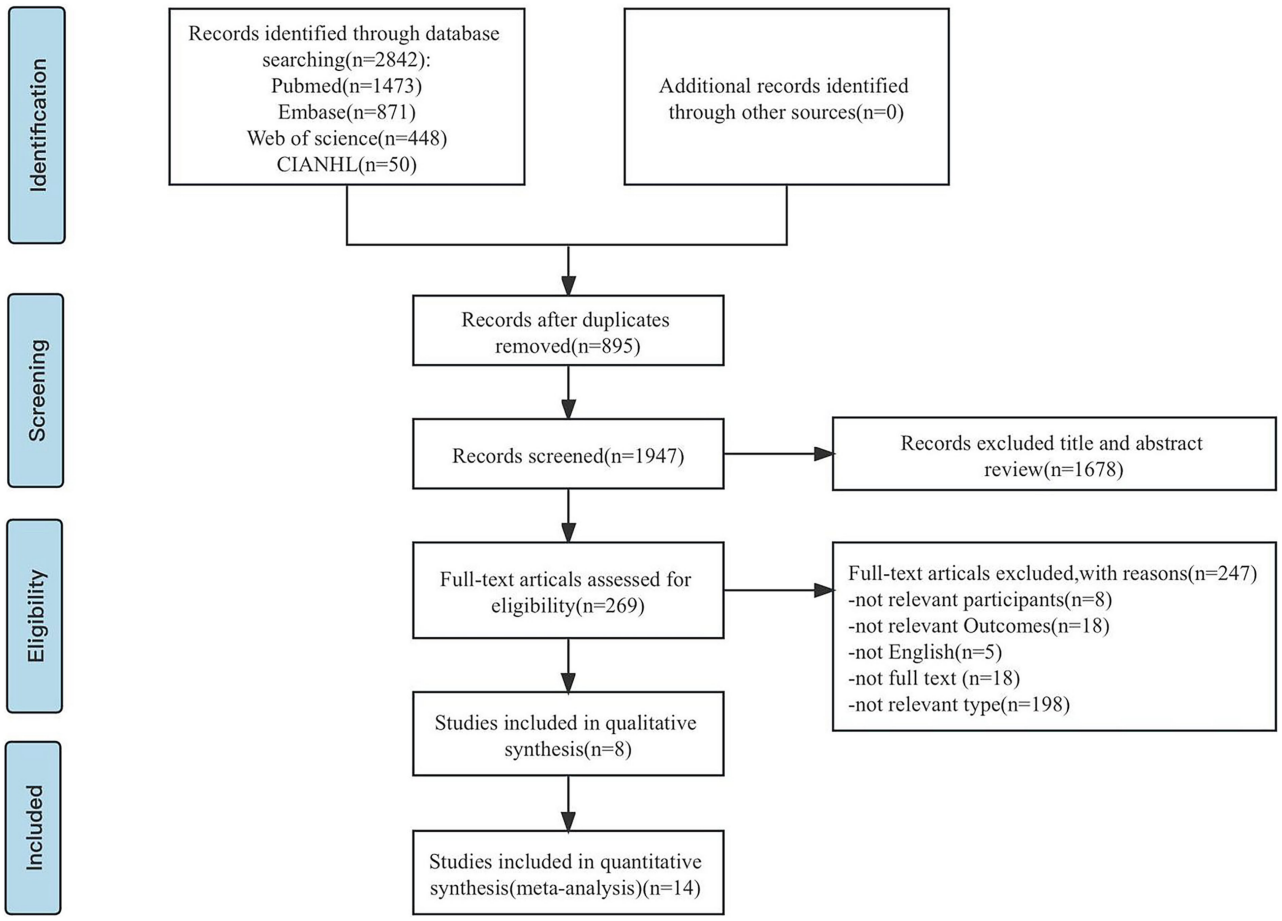
PRISMA flowchart: overview of in- and exclusion process. n, number.

The selection process is illustrated in Figure 1. According to inclusion and exclusion criteria, the literature will be screened by two researchers independently, compiled the data and verified it. Import all the retrieved documents into Endnote 20 software to remove duplicate documents, and two researchers read the title and abstract of the remaining literature to make a preliminary selection. The full text of the literature was screened again after the initial screening, to determine the final inclusion of literature. The following information was extracted from the included literature: author, date of publication, country, sample size, type of study, age, risk factors involved, etc. A third party could decide whether controversial literature would be included in the literature selection process.

### 2.4 Quality appraisal

The quality of the literature was evaluated by two researchers independently. The cross-sectional study’s quality was assessed by the US Agency for Healthcare Research and Quality using a set of 11 items, with responses categorized as “yes,” “no” and “unclear.”

Out of a total score of 11, studies with a score of 0–3, 4–7, and 8–11 were classified as low, medium, and high quality, respectively. The cohort studies and case–control studies were evaluated by the Newcastle-Ottawa Scale recommended by the Cochrane Collaboration.NOS included 3 dimensions: Selection, Comparability and Outcome, with a total of 8 items, full score of 9 points. If the score was greater than 7, the literature was considered to be of high quality, on behalf of the low quality of literature conversely. Where there is a dispute between the two opinions, they shall be resolved by negotiation, and if there is still disagreement after consultation, a third party decision will be sought.

### 2.5 Statistical analysis

Meta-Analyses were performed when there were at least three studies. The effect was characterized using the odds ratio (OR) and a 95% confidence interval (CI). For the influencing factors where only the mean and standard deviation were reported, the equation (21) was used to convert them into RoM values and their 95%CI. If only the standardized regression coefficient *β* and standard error were reported in the literature, the equation (22) was used to convert them into OR value and 95%CI.

In hetero-geneity inspection methods use Q combined with I2 quantitative judgment. When *p* > 0.05, *I*^2^ < 50%, it suggests that the heterogeneity among studies was deemed acceptable, and the fixed effect model was employed for consolidation analysis. The random effect model was employed for analysis if *p* ≤ 0.05, *I*^2^ ≥ 50% indicated that there was significant heterogeneity between the trials. Descriptive analyses were used if heterogeneity was too large and could not be resolved, or if the source of heterogeneity could not be identified.

Meta-regression analysis was conducted to investigate the potential sources of heterogeneity, and subgroup analysis was performed if factors were detected as being significant in a meta-regression. Sensitivity analysis was used to test the stability of meta-analysis results. Publication bias was evaluated using a funnel plot, Begg’s test and Egger’s test. A meta-analysis was performed with Stata16.0 software. A two-sided *p* < 0.05 was considered to be statistically significant.

## 3 Results

### 3.1 Study selection

A total of 2,842 relevant studies were retrieved. The software Endnote 20 was used to import all of the recovered literature. After deleting the duplicate research, a total of 1947 relevant studies were found. After reviewing the study titles and abstracts, 1,678 articles were rejected based on the inclusion and exclusion criteria of this investigation. After re-reading the full text of the literature that might meet the inclusion criteria, 247 studies were excluded, and 22 studies were finally included.

Included in the study, 21 items for the cross-sectional study, 1 for the cohort study. Figure 1 depicts the precise procedure and outcomes of the literature screening.

### 3.2 Characteristics and quality of included studies

A total of 22 articles (23–44) were included, published from 2004 to 2022, involving 89,897 subjects from 10 countries, including the United States, China, and Iran. The type of sleep disorder in perimenopausal women investigated in these 22 studies was insomnia. Following an extensive screening process, AHRQ rated 21 cross-sectional studies (23, 24, 26–44) as medium quality literatures with methodological quality evaluation ratings of ≥5 points. One cohort study(25)that NOS examined received an 8 on the quality rating scale, indicating that it was high-quality literature. See Table 2 for the basic characteristics and methodological quality evaluation of the included literature. The fundamental traits and methodological quality assessment of the included literature are listed in Table 2.

**Table 2:** Basic characteristics of the included studies.

First author	Country	Year	Sample size	Research type	Influencing factors	Quality score (points)	
						NOS	AHRQ
E. W. Freeman	United States	2015	436	Cohort study	F1, F4	8	
M. Arakane	Ecuador	2011	340	Cross-sectional study	F1, F2, F3		7
K. E. Ensrud	United States	2009	217	Cross-sectional study	F1		7
M. Gopal	United States	2008	300	Cross-sectional study	F14, F19, F20, F21		7
P. Chedraui	Ecuador	2013	204	Cross-sectional study	F1, F2, F22, F23, F26		7
Q. Zhou	China	2021	467	Cross-sectional study	F1, F8, F14, F39		7
Y. C. Chang	China	2010	85	Cross-sectional study	F2, F5, F7, F8, F9		6
F. Ahmady	Iran	2022	323	Cross-sectional study	F10, F11		6
H. C. Hsu	China	2005	197	Cross-sectional study	F10, F12, F17, F32, F43		6
M. J. Kim	South Korean	2018	634	Cross-sectional study	F8, F15, F16, F24, F25		6
M. Tadayon	Iran	2019	252	Cross-sectional study	F25, F26		6
R. Andenæs	Norway	2020	996	Cross-sectional study	F2, F13, F14, F25, F29, F30		6
S. M. Valiensi	Argentina	2019	195	Cross-sectional study	F1, F20, F31, F32		6
J. D. Kloss	United States	2004	168	Cross-sectional study	F8, F14, F16		6
J. E. Blümel	Latin American	2012	6,019	Cross-sectional study	F2, F5, F8, F10, F12, F13, F14, F15, F16		5
J. H. Hwang	South Korean	2021	3,000	Cross-sectional study	F7, F8, F12, F13, F17, F18		5
M. D. Zambotti	United States	2014	222	Cross-sectional study	F1		5
S. A. Creasy	United States	2019	75,074	Cross-sectional study	F3, F27, F28		5
S. Taavoni	Iran	2015	700	Cross-sectional study	F10, F15, F24, F33, F34, F35, F36, F37, F38		5
M. F. Naufel	Brazil	2018	53	Cross-sectional study	F6, F20, F40, F41, F42		5
M. D. Zambotti	United States	2015	33	Cross-sectional study	F8, F14		5
C. Seib	Australia	2014	322	Cross-sectional study	F3, F10, F12, F25, F26, F33, F41, F43, F44		5

Risk factor: F1: hot flash; F2: psychotropic drug use; F3: sedentary lifestyle; F4: mild/severe poor sleep in premenopause; F5: hormone intake; F6: hip circumference; F7: nutritional supplement intake; F8: depression; F9: perimenopausal fatigue; F10: age; F11: body mass; F12: chronic disease; F13: alcohol use; F14: anxiety; F15: educational level; F16: vasomotor symptoms; F17: postmenopausal states; F18: marital status; F19: race; F20: obstructive sleep apnea symptoms; F21: nocturia; F22: male premature ejaculation; F23: parity positively; F24: income; F25: physical health; F26: mental health; F27: physical activity; F28: time spent in light; F29: tobacco use; F30: satisfaction with life; F31: leg spasm; F32: snoring; F33: occupation; F34: husband’s profession; F35: number of children; F36: partner’s age; F37: consumption of tea, coffee or cola;F38: family size; F39: sweating; F40: waist circumference; F41: BMI; F42: waist-to-hip ratio; F43: overall self-rated activity levels; F44: number of menopausal symptoms.

### 3.3 Results of meta-analysis

Among the 22 studies, 12 studies (23–34)could be included in the quantitative analysis. A total of 6 influencing factors that could be extracted and analyzed by meta-analysis, including hot flashes, psychotropic drug use, depression, chronic disease, mental health and physical health. Their OR values and 95%CI were pooled for analysis. According to the results of the meta-analysis, there was no significant association found between the mental and physical health of perimenopausal women and sleep problems; instead, hot flashes, psychotropic drug usage, depression, and chronic disease were the characteristics that influenced the sleep disorders of these women.

Women with depression, hot flashes, chronic medical conditions, and psychotropic drug use during the perimenopausal periods have a significantly increased risk of sleep disorders, which are 2.73 times, 2.70 times, 1.39 times, and 3.19 times higher, respectively, than those without abnormal conditions during the perimenopausal periods. However, physical health and mental health were not significantly related to the occurrence of sleep disorders in perimenopausal women (see Table 3 for details).

**Table 3:** Meta-analysis results of influencing factors of perimenopausal women.

Influencing factors	Heterogeneity test		Effects model	Meta-analysis results				Number of studies
	*I*^2^(%)	*p*		*Z*	*p*	OR	95%CI	
Depression	95.4	<0.001	Random-effects model	3.89	<0.001	2.73	1.65 ~ 4.52	4
Hot flashes	83.8	<0.001	Random-effects model	4.85	<0.001	2.70	1.81 ~ 4.02	5
Chronic disease	0	0.73	Fixed-effects model	5.64	<0.001	1.39	1.24 ~ 1.56	3
Psychotropic drug use	96.6	<0.001	Random-effects model	2.56	0.01	3.19	1.31 ~ 7.77	3
Mental health	97.3	<0.001	Random-effects model	1.04	0.30	0.87	0.66 ~ 1.14	3
Physical health	98.6	<0.001	Random-effects model	0.99	0.32	1.73	0.59 ~ 5.06	3

### 3.4 Descriptive analysis

Only a descriptive analysis will be done for these influencing factors since effective data cannot be recovered for a quantitative combination of some of them or because only one piece of literature was reported on them.

Three articles (23, 33, 38) found that among women going through menopause, a sedentary lifestyle increased their likelihood of developing sleep disturbances. According to five research (26, 33–35, 44), perimenopausal women’s age was associated with an increased risk of sleep disturbances. Alcohol use was linked to three different risk factors (26, 27, 37). Anxiety was considered as a risk factor in 5 articles (26, 37, 39, 40, 42). Three studies (26, 30, 39) proposed that vasomotor symptoms were the risk factors, while three more regarded the symptoms of obstructive sleep apnea to be risk factors (36, 41, 43).

Some studies suggest that poor sleep in premenopause (25), body mass (35) marital status (27), leg spasm (43), snoring (34), sweating (42) and number of menopausal symptoms (33) were risk factors for sleep disorders in perimenopausal women. Other studies have also pointed out that hormone intake (24, 26) nutritional supplement intake (24, 27), perimenopausal fatigue and states (24, 27, 34, 41) are risk factors for sleep disorders in perimenopausal women.

Gopal et al. (36) suggested that race and nocturia were risk factors for sleep disorders in perimenopausal women. Chedraui et al. (32) suggested that male premature ejaculation is a risk factor and parity positively is a protective factor for sleep disorders in perimenopausal. Creasy et al. (38) reported that physical activity was a protective factor and time spent in light was a risk factor for sleep disorders in perimenopausal. Andenæs et al. (37) reported that satisfaction with life was a protective factor for sleep disorders in perimenopausal women, while tobacco use was a risk factor. Taavoni et al. (44) suggested that husband’s profession was a protective factor for perimenopausal sleep disorders. Number of children, partner’s age, consumption of tea, coffee or cola and family size were risk factors.

In the study by Naufel et al. (41), the linear regression model revealed that the wake after sleep onset (WASO) (the amount of time spent awake during the sleep period) and waist circumference were significantly positively connected, while the rapid eye movement sleep latency (LREM) (the number of minutes after sleep onset to enter the rapid eye movement sleep stage) was positively correlated with BMI and hip circumference. It can be said that postmenopausal women who have high body mass indexes and abdominal obesity have a harder difficulty going into the rapid eye movement sleep stage (REM), increase night awake time, and reduce deep sleep and sleep efficiency, which can result in insomnia. Thus high BMI and abdominal obesity are risk factors for sleep problems in perimenopausal women. The waist-to-hip ratio has a negative correlation with sleep efficiency, so a low waist-to-hip ratio is a protective factor.

In addition, some studies suggested that educational level (44), occupation (33, 44) overall self-rated activity levels (33, 34) and income (30, 44) were protective factors for sleep disorders in perimenopausal women.

### 3.5 Sensitivity analysis

The meta-analysis results’ stability was examined by excluding papers with a high weight or poor quality, and by using the conversion effect model. Every influencing element underwent a separate sensitivity analysis. The results of the meta-analysis were steady and trustworthy if the examination of each influencing component before and after the analysis was essentially consistent. Table 4 provides further information.

**Table 4:** Sensitivity analysis results.

Influencing factors	Before sensitivity analysis		After sensitivity analysis		Stability
	OR (95%CI)	*p*	OR (95%CI)	*p*	
Depression	2.73 (1.65 ~ 4.52)	<0.001	2.33 (2.13 ~ 2.57)	<0.001	Stable
Hot flashes	2.70 (1.81 ~ 4.02)	<0.001	2.61 (2.28 ~ 3.00)	<0.001	Stable
Chronic disease	1.39 (1.24 ~ 1.56)	<0.001	1.39 (1.24 ~ 1.56)	<0.001	Stable
Psychotropic drug use	3.19 (1.31 ~ 7.77)	0.01	3.22 (2.91 ~ 3.57)	<0.001	Stable
Mental health	0.87 (0.66 ~ 1.14)	0.30	0.89 (0.88 ~ 0.91)	<0.001	–
Physical health	1.73 (0.59 ~ 5.06)	0.32	2.80 (2.53 ~ 3.09)	<0.001	–

### 3.6 Publication bias

There was no discernible publication bias, according to the results of the Egger’s and Begg’s tests performed with Stata16.0 software. For more information, see Table 5.

**Table 5:** Test for publication bias of influencing factors of perimenopausal women.

Influencing factors	Egger’s test		Begg’s test	
	*t*	*p*	*Z*	*p*
Depression	0.51	0.663	0.34	0.734
Hot flashes	0.15	0.888	0.24	0.806
Chronic disease	1.66	0.345	1.04	0.296
Psychotropic drug use	−0.23	0.858	0.00	1.00
Mental health	0.27	0.832	0.00	1.00
Physical health	−1.75	0.331	0.00	1.00

## 4 Discussion

### 4.1 Main working factors

The relationship between sleep disorders and female reproductive aging is still unclear. According to the US SWAN study statistics (45), perimenopausal women have a higher risk of insomnia than reproductive women; Kravitz et al. and Ciano et al. also reported a higher prevalence of sleep disorders during the menopausal transition (11, 46). The reason for the association of sleep disturbances with menopause in these studies may be the fluctuating levels of sex hormones in women during this period, which are known to regulate sleep (47). Woods et al. reported that sleep disorders are more severe in postmenopausal women than in reproductive women (48). Additionally, Xu (49) and Lampio (16) have suggested that perimenopausal women are more likely than reproductive-aged women to experience sleep disturbances. Nevertheless, some writers contend that sleep issues are only strongly linked to the menopausal transition and not the postmenopausal period (45, 50). A well-known epidemiological study found that menopause does not worsen sleep quality (51). Some earlier studies also found that the prevalence of severe sleep disorders did not increase significantly or only slightly with advancing age (52–54).

In perimenopausal women, depression is a significant contributing factor to sleep problems, as demonstrated by this study. Other research has indicated that severe depressive episode or depressive state independently influences sleep disorders (55). Furthermore, perimenopausal women’s reports of sleep disruptions were regularly and strongly correlated with depression, according to research by R. L. Smith et al. (14). Other studies have also suggested that depressive symptoms may be a major contributor to sleep problems (56, 57). In the meantime, women in today’s society face increased pressure from their families, careers, and society at large. Additionally, because perimenopausal women experience more changes in their professional lives and are more sensitive to emotions, their anxiety rates are significantly higher than those of women who are childbearing age. In addition to physiological factors, cognitive factors, personality traits, occupational factors, economic status, social support and other aspects may contribute to the heavier psychological load borne by perimenopausal women, leading to their depression and anxiety. Previous studies have found a close relationship between psychological activity and physiological conditions, with prolonged anxiety predisposing to somatic disorders (58), mood disorders being a strong predictor of sleep disorders (59), and a possible bidirectional relationship between sleep disorders and depressed mood (60).

According to this study, hot flash-afflicted perimenopausal women were more prone to report sleep problems. One of the causes of perimenopausal sleep disorders was found to be hot flashes, which were reported to be responsible for insomnia in 80% of perimenopausal women (61). Hot flashes, which can start in the late 30s or early 40s, are frequently linked to insomnia and worse sleep quality (62). It has also been shown that 72.2% of perimenopausal women have sleep disorders, of which 39.8% have poor sleep quality, suggesting that hot flashes are significantly associated with sleep disorders (63). However, the exact mechanism by which hot flushes affect sleep is still unclear. Some scholars have suggested that the increase in sleep disorders is due to the decline in estrogen during perimenopause and postmenopause, leading to the development of menopause-related symptoms such as vasoconstriction, which in turn affects sleep at night, and some scholars have suggested that estrogen is beneficial in maintaining circadian rhythms of sleep and improving the stability of sleep.

The quality of sleep varies among perimenopausal women with different health conditions. When perimenopausal syndromes are combined with chronic illnesses like diabetes, hypertension, and heart disease, it can cause significant physiological discomfort for some perimenopausal women. This discomfort can then translate into poor moods and sleep disturbances. Some studies have shown that the pain and discomfort associated with long-term chronic illnesses can affect the quality of sleep (64).

It has been proposed that women who struggle with poor sleep during the perimenopausal stage may consider taking nutritional supplements or prescription drugs to enhance their general health or quality of sleep (20), and that the use of medications, hormones, or nutritional supplementation intake may also result in their sleep quality being compromised. This finding is also consistent with previous studies (50). Therefore, there is a need to assess the types and side effects of medications being taken by perimenopausal women.

### 4.2 Other influencing factors

Some studies have shown that vasomotor symptoms may be a major contributor to sleep problem (57, 65). On the other hand, additional research has demonstrated that there is still uncertainty regarding the association between menopausal vasomotor symptoms and sleep disturbances and that vasomotor symptoms are not always the cause of sleep issues (29). Vasomotor symptoms and sleep disturbances were not linked, according to a study done on middle-aged Chinese women (56).

The majority of early research revealed a strong correlation between menopausal status and poor sleep quality (66, 67), as well as a relationship between sleep issues and both physical and psychological menopausal pause symptoms (68). Patients with notable menopausal symptoms had poorer quality sleep, according to a Turkish study (69).

A correlation between menopausal status and sleep disorders has also been documented in earlier research (70), These studies found that postmenopausal women were substantially more likely than premenopausal women to experience sleep disorders, and that menopausal status also increased the total PSQI score and a number of PSQI items, such as time to fall asleep, habitual sleep efficiency, and sleep disturbances. However, R. L. Smith et al. concluded that sleep quality in women is not related to menopausal status (14).

Although the exact causes of menopausal sleep disturbances are unknown, a variety of factors, such as vasomotor symptoms, hormonal fluctuations, mood disorders, comorbid diseases, and lifestyle choices, may be at play (71). Reduced hormone levels, in particular, might be a major factor in sleep disturbances (72–75). The Wisconsin Sleep Cohort Study, which included 589 premenopausal women in pause, perimenopausal, and postmenopausal women, found that postmenopausal women had the best sleep, while sleep worsened in women taking hormone replacement therapy (51). And in that cohort study, no evidence was found that hot flashes caused sleep disturbances.

According to the current study, obesity may also have a detrimental effect on sleep quality. A high body mass index (BMI) has been linked to higher respiratory resistance and partial upper airway obstruction (64, 76),both of which may cause poorer sleep quality in perimenopausal women who are heavier. In addition, smoking is a risk factor for menopausal sleep disorders, a finding that is also shared with previous studies (49). Additionally, R. L. Smith discovered (14) that smoking was still associated with sleep problems and insomnia in perimenopausal women, even after controlling for vasomotor symptoms and hot flashes. And lastly, research indicates that women with lower incomes and educational levels are more likely to experience sleep difficulties (26, 70). Thus, there may be a favorable effect of higher levels of education and income on sleep quality.

### 4.3 Strengths and limitations

This study indicates that there is a need for thorough evaluation, treatment, and education of women going through the menopausal transition. It also emphasizes prevalent risk factors linked to sleep disturbances in perimenopausal women.

However, there are some limitations in this study: ① Even though this study created a strict search strategy and carried out an exhaustive search, it only looked through English-language literature, and only a small number of articles were eventually included, which would have affected how thorough the results were.② Confounding variables may have influenced the meta-analysis’s findings, given the majority of the 14 included papers were cross-sectional studies and only one was a prospective cohort study;③ Large differences in sample size and assessment tools between studies may be a source of bias;④ The results could be affected in some way by the exclusion of some of the literature that could perform risk factor analyses;⑤ The results’ correctness is impacted by the connected influencing factors. Prospective cohort studies with high sample sizes and multicenter design should be carried out in the future to assess the factors that influence sleep disturbances in women going through menopause. This will ensure that the research findings are thorough and supported by science.

### 4.4 Implications for practice

Regarding the possible influence of hormonal treatment, vasoconstrictor symptoms and hot flashes on sleep disruptions in perimenopausal women, this study did discover some disagreement. According to the majority of research, all three are linked to sleep issues in women going through menopause. Since the effects of hot flush on perimenopausal women are unclear, this discrepancy could be due to a variety of factors, including the study design, sample size, subjective factors during the investigation, individual hormone tolerance, participant race, comorbidities, physical health status, living situation, and other confounding variables. Therefore, while assessing sleep disturbances in perimenopausal women, individual characteristics must be taken into account. Since various people may exhibit different symptoms, it’s important to thoroughly assess perimenopausal women’s sleep health from a range of angles. Second, in order to homogenize the research objects and better assess the influencing aspects, confounding variables should be minimized in the inquiry and the research objects should be rigorously screened.

Women should be instructed about sleep hygiene, such as bed-only sleeping and sexual behavior (77), and getting up if women are unable to sleep. Also, reduce daytime naps, reduce intake of caffeinated beverages, and receive health education for life stressors. For perimenopausal women, a comprehensive evaluation of sleep disorders is needed in conjunction with medical history, sleep diary, questionnaire assessment, and objective assessment of sleep with polysomnography if necessary. Every woman who is about to enter the perimenopause should be educated about the signs and symptoms of menopause, as well as the health risks associated with smoking, depression, anxiety, medication use, chronic illnesses, and sleep disorders. Additionally, women who report having a sleep disorder should be assessed for these conditions as well as for smoking, chronic illness, and depression.

Relevant institutions may also set up perimenopausal women’s outpatient clinics, establish perimenopausal women’s handbooks and health cards, and regularly follow up and monitor the status of perimenopausal women; perimenopausal women’s health education campaigns are carried out on a regular basis in community health service institutions, outpatient clinics and so on; set up a perimenopausal women’s social support network, so as to understand perimenopausal women’s physiological and psychological changes, and provide guidance and psychological support to perimenopausal women; guide perimenopausal women to establish a healthy lifestyle, adjusting their dietary structure, and cultivating good dietary habits, and guiding perimenopausal women to carry out reasonable sports and exercise in the light of their own actual conditions to improve their physical fitness; They should also be instructed to learn self-regulation and do a good job of psychological care, meaning that they should keep a correct mindset to face negative events in life and work.

Currently, estrogen therapy is the best pharmacological treatment for women with menopausal symptoms, especially for those with severe hot flashes (64). Although other medical treatments such as melatonin, sedatives and hypnotics, and antidepressants may provide relief for menopausal insomnia, the side effects of long-term use should be carefully considered. For women with perimenopausal sleep disorders, the effects of organism aging itself on brain function and sleep should also be considered comprehensively. Taking into account the patient’s specific situation, multidisciplinary collaborative diagnosis and treatment should be carried out to provide individualized treatment and nursing plan.

## 5 Conclusion

Sleep disturbance is one of the most common symptoms and a common problem for perimenopausal women seeking care. With the transition from fertility to menopause, perimenopausal sleep disorders are triggered by multiple factors such as changes in female sex hormones, hot flashes and sweating, mood disorders, and changes in the circadian rhythm biological clock.

This study suggests that the occurrence of perimenopausal sleep disorders is associated with depression, hot flashes, health conditions and psychotropic drug use, which is therefore beneficial for women and medical professionals to gain a better understanding of the risk factors for the occurrence of perimenopausal sleep disorders and to take appropriate measures to prevent and manage perimenopausal symptoms, and to face the female reproductive aging with a positive mindset.

Healthcare providers need to be aware of how sleep disorders affect women’s health and quality of life, perform a thorough assessment of their menopausal symptoms, and assist them in selecting the psychological, environmental, and pharmacological perimenopausal management options that are best for them.

Currently, there is controversy as to whether hormone replacement therapy hot flashes and vasoconstrictive symptoms have an effect on sleep quality in perimenopausal women, and further studies are needed by researchers. Due to the large variations in sample sizes and heterogeneity among the studies included in this study, large-sample, multicentre prospective studies are still needed in the future.
